# Andrographolide-Loaded Gold Carbon Quantum Dots and Their Doped Derivatives for Enhanced Hydrophilicity in a Drug Delivery System

**DOI:** 10.3390/pharmaceutics18060647

**Published:** 2026-05-24

**Authors:** Wenndy Pantoja-Romero, Alexis Lavín Flores, Alejandro Lozada-Jerez, MiaSara Perez-Salvá, Fabiola Rosa-Suárez, Orestes Quesada, Magaly Martínez-Ferrer, Gerardo Morell, Brad R. Weiner

**Affiliations:** 1 Department of Chemistry, University of Puerto Rico, Rio Piedras Campus, San Juan, PR 00925, USA; alexis.lavin@upr.edu; 2 Molecular Sciences Research Center, University of Puerto Rico, San Juan, PR 00925, USA; miasara.perez@upr.edu (M.P.-S.); fabiola.rosa3@upr.edu (F.R.-S.); orestes.quesada@upr.edu (O.Q.); gerardo.morell@upr.edu (G.M.); 3 Faculty of Health and Human Wellbeing, School of Dentistry, Indoamerica Technological University, Ambato 180202, Ecuador; joselozada@uti.edu.ec; 4 Department of Biology, University of Puerto Rico, Rio Piedras Campus, San Juan, PR 00925, USA; 5 Department of Physical Sciences, University of Puerto Rico, Rio Piedras Campus, San Juan, PR 00925, USA; 6 Department of Pharmaceutical Sciences, School of Pharmacy, University of Puerto Rico, Rio Piedras Campus, San Juan, PR 00925, USA; magaly.martinez1@upr.edu; 7 Division of Cancer Biology, University of Puerto Rico Comprehensive Cancer Center, San Juan, PR 00925, USA; 8 Department of Physics, University of Puerto Rico, Rio Piedras Campus, San Juan, PR 00925, USA

**Keywords:** gold nanocomposites, carbon-based quantum dots, doped-carbon quantum dots, nanocarriers, cell viability, morphological studies in human red blood cells

## Abstract

**Background/Objectives:** Andrographolide (ADG) is a plant-derived compound with promising anticancer properties, but its medical use is limited due to poor water solubility and low bioavailability. This study proposes developing a gold-based nanocomposite drug delivery system, using a simplified synthesis method, to improve ADG’s hydrophilicity and enhance its delivery efficiency. **Methods:** A one-step method was used to synthesize gold nanocomposites with carbon quantum dots (CBQDs) and doped CBQDs acting as reducing and stabilizing agents. These nanocomposites were then conjugated with ADG and thoroughly characterized using multiple structural and spectroscopic techniques such as X-ray diffraction (XRD), Fourier transform infrared spectroscopy (FTIR), ultraviolet–visible spectroscopy (UV–Vis), transmission electron microscopy (TEM), Raman spectroscopy, and nuclear magnetic resonance (NMR) spectroscopy. Hydrophilicity enhancement was evaluated using NMR-based log P measurements. Biological assessment involved cell viability assays and confocal microscopy studies in PC3 prostate cancer cells, along with the morphological evaluation of human red blood cells. **Results:** XRD confirmed the formation of crystalline, face-centered cubic gold nanoparticles, while spectroscopic analyses verified successful nanocomposite formation and ADG conjugation. NMR results showed enhanced hydrophilicity of ADG. Biological tests demonstrated that the nanocomposites were compatible with cells. **Conclusions:** This study presents a straightforward strategy for synthesizing gold-based nanocomposites that enhance the hydrophilicity and delivery potential of andrographolide, supporting their applicability as nanocarrier platforms for anticancer drug delivery.

## 1. Introduction

Advances in drug delivery technologies have paved the way for numerous pharmaceutical innovations aimed at improving patient health. These innovations optimize therapeutic delivery to target sites, minimize off-target accumulation, and improve patient compliance [1,2]. The choice of a drug delivery method greatly influences the effectiveness of the medication. Utilizing an appropriate drug delivery system can enhance various crucial pharmacological properties of the drug that may not be optimized when administered in its free form [3]. Multifunctional nanomedicine therapeutics have been developed by combining drug delivery and responsive systems. These systems have been used in phase 1 clinical trials for patients with solid tumors [4]. However, recent advances in nanomaterial synthesis have led to the development of new drug systems that enable multiple therapeutic modalities within a single carrier. Among them, various multifunctional nanomaterials such as gold nanoparticles, semiconductor quantum dots, carbon nanomaterials, and magnetic nanoparticles have been explored for theranostic applications [5].

Carbon-based quantum dots (CBQDs) are small carbon nanoparticles that not only have the excellent physicochemical properties of metallic quantum dots, such as stability and tunable fluorescence, but also benefit from easy synthesis, high biocompatibility, and low toxicity [6]. CBQDs have gained interest in the biomedical field and show great potential to significantly impact cancer therapy and diagnosis [7].

Pyrolysis is an environmentally friendly and facile method for preparing CBQDs, which uses suitable organic molecules or polymers for dehydration and further carbonization [8,9]. Doping CBQDs with heteroatoms (such as sulfur (S) and nitrogen (N)) modulates the bandgap, alters electronic properties, and enhances performance in biomedical applications [10,11]. These dopants introduce new electronic energy levels that facilitate incorporation into the carbon lattice. Nitrogen’s smaller atomic size and higher electronegativity facilitate substitution into the carbon lattice, introducing n-π∗ transitions that enhance photoluminescence quantum yield (PLQY) by reducing non-radiative decay pathways [12,13]. Doping nitrogen and sulfur into CBQDs is particularly advantageous for drug delivery systems (DDSs) explored in this study. Compared with other dopants such as phosphorus, boron, or various metals, nitrogen and sulfur simultaneously enhance drug loading capacity, control release, and biocompatibility, which are key requirements for an effective nanocarrier. In contrast, other dopants often require additional chemical modification steps, offer less versatile surface chemistry, show weaker responsiveness to biological stimuli, and may introduce toxicity concerns [14,15].

Decorating CBQDs and doped-CBQDs (D-CBQDs) with metal atoms can modify their optical, chemical, and electronic properties, enabling these materials for a variety of applications [16]. In this sense, the use of gold nanoparticles (AuNPs) is a good candidate to decorate the CBQDs. AuNPs have optical and electronic properties such as conductivity, localized surface plasmon resonance (LSPR), stability towards oxidation, biocompatibility, and catalytic activity, which can be adjusted by changing their size, shape, surface chemistry, and aggregation state [17,18]. In addition, AuNPs exhibit anticancer activity through various mechanisms, depending on their metal properties, and can be used in chemo-photothermal therapy or photothermal therapy [19,20].

The interaction of AuNPs with carbon-based nanomaterials has been reported to prevent the aggregation of AuNPs [21]. A multitude of physical and chemical techniques exist for producing gold nanoparticles. Among these methods, the reduction in chloroauric acid (HAuCl_4_) using various reducing agents such as sodium citrate, sodium borohydride, hydrazine, and dimethyl formamide stands out as a commonly employed approach for gold nanoparticle synthesis [22,23].

Andrographolide (ADG) is an ent-labdane diterpenoid principally used to treat prostate cancer (PC). It has high lipophilicity (log P value = 2.632 ± 0.135) and low aqueous solubility (3.29 ± 0.73 μg/mL) [24]. PC is the most common cancer among men in the United States, with one in five men receiving a diagnosis [25]. It can be classified as androgen-sensitive or androgen-insensitive, depending on its response to testosterone stimulation, which also influences treatment options. The main goal of endocrine therapy for prostate cancer is to eliminate the stimulating effect of androgen on prostate cancer cells. This can be achieved using drugs such as GnRH-agonists or antagonists and non-steroidal antiandrogens [26,27,28]. These therapies come with significant drawbacks, including nonspecific toxicity, multidrug resistance, and a high recurrence rate, creating challenges to achieve satisfactory clinical outcomes [29]. Additionally, many of these drugs have several side effects. Therefore, developing an effective DDS that can provide potent and targeted cancer therapy is of great interest to both the scientific and healthcare communities.

Recent research on ADG delivery has increasingly focused on overcoming its poor solubility and low bioavailability through the development of advanced nanocarrier systems. Lipid-based platforms, including solid liquid nanoparticles (SLNs), nanostructured lipid carriers (NLCs), and liposomes, are among the most extensively studied due to their ability to enhance drug stability, bioavailability, and targeted delivery, while niosomes have emerged as cost-effective alternatives [30]. In parallel, polymeric and protein-based nanoparticles offer controlled release profiles and improved biocompatibility. For oral administration, self-microemulsifying drug delivery systems (SMEDDSs) and nanoemulsions have demonstrated significant improvements in dissolution and intestinal absorption [31]. More recently, advanced platforms such as core–shell nanoparticles and gold-based nanocarriers have enabled multifunctional approaches, including targeted and stimuli-responsive delivery. For instance, Assim et al. developed an SMEDDS formulation composed of Capryol PGMC, Tween 20, PEG 300, and Transcutol HP, selected for their ability to improve the solubility of ADG. This system significantly improved solubility, dissolution rate, plasma exposure, and overall therapeutic efficacy, highlighting its potential for oral delivery applications [32]. Similarly, Tipduangta et al. employed solid dispersion (SD) techniques using polymer matrices such as PEG, Soluplus, PVP, PVP-VA 64, HPMC, and Poloxamer 188. Their results showed a marked enhancement in ADG solubility, achieving up to a 4.7-fold increase, along with a substantial reduction in drug release half-time (T_1/2_) to less than 5 min, indicating a faster and more efficient drug release profile [33].

In this context, we have devised a one-step procedure for synthesizing AuCBQDs (gold–carbon quantum dots) and AuD-CBQDs (gold-doped carbon quantum dots) nanocomposites. Given carbon’s efficacy of reducing Au^3+^ to AuNPs, CBQDs, and D-CBQDs were utilized as the reducing agents [34]. These synthesized nanomaterials were then conjugated with ADG to address its poor solubility and permeability. We expect that this conjugation will enhance ADG’s bioavailability and therapeutic efficacy against prostate cancer cells [35]. Understanding the hydrophilicity of nanoparticles is essential for evaluating their potential in drug delivery. This has been explored by analyzing the partition coefficient through NMR spectroscopy, a technique previously used to assess CBQDs and D-CBQDs [36]. Additionally, cell viability in PC3 prostate cancer cells and confocal microscopy were examined to gain insights into cancer cell behavior and their response to treatments with nanocomposites. Morphological studies of human red blood cells (RBCs) serve as a vital tool for investigating various physiological and metabolic processes. RBCs contain hemoglobin (Hb), a red-colored molecule that constitutes approximately 33% of their intracellular content [37,38]. To the best of our knowledge, no prior quantitative studies have examined the effects of andrographolide-loaded nanocomposites on RBCs.

## 2. Materials and Methods

### 2.1. Chemicals

Citric acid (CA), 3-mercaptopropionic acid (MPA), ammonium chloride (NH_4_Cl), sodium hydroxide (NaOH), chloroauric acid (HAuCl_4_), deuterium oxide (D_2_O) 99.95 atom % D, and 1-octanol ≥98%, were acquired from Sigma Aldrich (Saint Louis, MO, USA) and Fisher Scientific (Cayey, PR, USA) and were used without further purification. Before use, all glassware was triple rinsed with double-distilled water. All Solutions were prepared utilizing deionized water by Aries Filter Works Gemini model GMS-105 with GMA-UV (West Berlin, NJ, USA), phosphate-buffered saline (PBS; Sigma Aldrich), and ethanol.

Nanopure water was obtained from an ARIES Filter Works Gemini high-purity water system (18.23 M-Ohm/cm; West Berlin, NJ, USA). PBS (tablets), RPMI Medium, Penicillin–streptomycin (Pen-Strep), fetal bovine serum (FBS), trypsin, trypan blue, glycerol, and formaldehyde were purchased from Sigma-Aldrich (Saint Louis, MO, USA). Human prostate cancer PC3 (ATCC^®^ CRL-1435™) was purchased from ATCC (Manassas, VA, USA). This product is intended for laboratory research use only.

### 2.2. Synthesis of CBQDs and D-CBQDs

CBQDs and D-CBQDs were synthesized as we reported previously [36].

### 2.3. Synthesis of Au-CBQDs and Au-D-CBQDs Nanocomposites

Au-CBQDs and doped-Au-CBQDs (S-CBQDs and N-CBQDs) were prepared through a one-step process using CBQDs and doped-CBQDs as the reducing agent. A stock solution of 150 μM HAuCl_4_ was added to the CBQDs and D-CBQDs solutions in a molar ratio of 1:1. The color of the mixture changed from yellow to pink after 30 min of incubation at 50 °C, indicating the formation of spherical AuNPs.

### 2.4. Coupling of ADG with Au-CBQDs and Au-D-CBQDs Nanocomposites

AuCBQDADG and AuD-CBQDs (AuSCBQD and AuNCBQD)-ADG were prepared using a one-step process. AuCBQDs and AuD-CBQDs were mixed with an ethanolic solution of ADG in a volume ratio of 1:1. The resulting nanocomposites were incubated in a sonicator at 50 °C for 30 min and then subjected to rotary evaporation. For maintaining the long-term stability of nanocomposites, the samples were frozen at −80 °C in containers filled to less than half their capacity, and subsequently loaded onto the collector at −45 °C under a vacuum of 0.133 mbar in a Labconco FreeZone 2.5 lyophilizer (Fort Scott, KS, USA).

### 2.5. Partition Coefficient Studies

The partition coefficient measurements were conducted using NMR spectroscopy following the methodology outlined by Cumming and Rücker [39]. The partition constant and its logarithmic value (c) can be assessed through partitioning in two immiscible solvents, generally water (D_2_O) and 1-octanol. The log P is calculated from the ratio of respective equilibrium concentrations of molecules in the two phases (Equation (1)).

(1) LogP=log(octanol/water)

The procedure involves initially dissolving a quantity of the compounds in D_2_O in an NMR tube, followed by the acquisition of a ^1^H NMR spectrum. Subsequently, an equivalent volume of 1-octanol is introduced into the NMR tube, and the phases are thoroughly mixed. The tube is then allowed to settle undisturbed to achieve phase separation and equilibrium. The resulting $K_{ow}$ (Equation (2)) serves as the partition constant, with its logarithmic value (log $K_{ow}$) regarded as the obtained log P.

(2) $$K_{\mathrm{ow}}=\frac{{RI}_{w}^{init}-{RI}_{w}^{equil}}{{RI}_{w}^{equil}},$$

where ${RI}_{w}^{init}$ represents the relative NMR signal integration of the materials in D_2_O before equilibration with 1-octanol, and ${RI}_{w}^{equil}$ denotes the relative NMR signal integration of the materials in the aqueous phase after equilibration.

### 2.6. General Solubility Equation (GSE) Calculations

Molar aqueous solubility (Sw) was estimated using the updated General Solubility Equation developed by Jain and Yalkowsky [40].

(3) $$LogS_{w}=0.5-0.01(MP-25)\;logK_{ow}$$

$S_{w}$ was determined and converted to μg/mL. The ADG utilized in this study possessed a melting point (MP) of 231 °C. The log K_ow_ denotes the partition coefficient (log P). For the GSE calculations, experimental log P values were obtained via NMR. At neutral pH, the ionization of ADG results in a log P value equivalent to its log D value (distribution coefficient) [41,42]. While log P represents the lipophilicity of neutral molecules, log D accounts for ionizable compounds; these values converge for non-ionizable species. Based on these data, ADG is characterized as an ionizable, lipophilic molecule.

### 2.7. Cytotoxicity Studies in PC3 Cancer Cells

Cell culture: Media with 0.05% Trypsin—0.53mM EDTA solution, diluted 1:1 with D-PBS, 0.1% Soybean Trypsin Inhibitor, or 2% fetal bovine serum in D-PBS were used to incubate PC3 cells at 37 °C and 5% CO_2_ in relative humidity. Cell passages were performed weekly between 80.0% and 90% confluency.

Cell seeding: PC3 cells were seeded in 96-well plates at a density of 7.0 × 10^4^ cells/mL. The 96-well plates were incubated at 37 °C in 5% CO_2_ in air for 24 h. Three 96-well plates were prepared per time point (48 h).

Cell treatments: Two-fold serial dilutions of AuCBQD, AuNCBQD, AuSCBQD (0–5000 µg/mL), AuCBQDADG, AuNCBQDADG, AuSCBQDADG (0–500 µg/mL), were prepared to determine the IC_50_. All treatments were performed after 24 h of cell seeding. In these experiments, cells were treated with 100 μL of each solution per well to determine the IC_50_. Cells were treated for 48 h. The control group was treated with media (RPMI or F-12, 1% Pen-Strep). All experiments were performed in triplicate.

MTS^®^ Assay: Cell proliferation was determined after 48 h of treatment. An MTS^®^ in RPMI solution was prepared depending on the number of plates to be incubated. Media was removed from all 96-well plates, and 100.0 μL of the MTS solution was added. The 96-well plates were incubated for 4 h before measurements. The fluorescence was measured at 560.0 nm of excitation and λ_max_ = 590.0 nm of emission. The percentage of live cells was achieved by taking into consideration the viability of the control group (100%) associated with the cells treated with ADG, unloaded, and loaded gold-nanocomposites.

### 2.8. Statistical Method

To determine the effectiveness of the proposed nanocomposites in cancer treatment, the concentration of nanocomposites at which only 50% of the cells survive is used as an indicator (IC_50_). A statistical dose–response (regression) analysis is needed to infer an IC_50_. A typical dose–response curve (DRC) that evaluates the effect of a drug on cancer cells is a curve with four parameters, with its X-axis corresponding to the concentrations of a drug (usually on a log scale), and its Y-axis corresponding to the drug responses.

(4) $$f(x)=c+\frac{d-c}{1+exp\left(b\left(\log x-\log e\right)\right)}$$

where f(x) is the drug response, x is the dose or concentration of a data point; c is the floor which shows the minimum cell activity at a high concentration of the chemical compound; d is the maximum cell activity which shows the biological activity without a chemical compound; e is the potency or the dose at which 50% of cell viability is obtained (IC_50_), and b is the slope. The open-source environment R program (Version 4.2.2, 2022) was used to evaluate the data obtained. This program offers a specialized sub-system for analysis of dose–response data through the add-on package drc (version 3.0.1, 2015). A statistical dose–response model is constructed using nonlinear regression applied directly to the raw, untransformed response data, which preserves its natural variance. But a comparison of dose–response curves (DRCs) across different experiments (e.g., cell lines or plates) is problematic due to variability in assay signal magnitude (absolute absorbance). Therefore, data must be normalized to a standard scale. A linear transformation of the response data does not alter the resulting IC_50_ estimates, ensuring a transformation to percentage of cell viability can be derived using the parameters established by the model fit to normalize the raw response data to a 0% to 100% scale. Cell viability is calculated using the following equation:

(5) $$\%Viability=\frac{{Response}_{sample}-Bottom}{Top-Bottom}\cdot 100$$

where Response_sample_ is the raw signal at a specific drug concentration. The top and bottom parameters are statistically determined from the nonlinear regression fit to define the 100% and 0% viability reference points, respectively. To avoid the pitfalls of a priori background correction, which may ignore background variability, a robust fitting method as suggested by Jarantow et al. [43] is employed, utilizing a Four-Parameter Logistic (4PL) model applied to the untransformed response data.

### 2.9. Model Fitting

The raw dose–response data Y (response signal) versus X (drug concentration) are fitted using the 4PL model to determine the Top and Bottom asymptotic parameters.

To ensure these parameters reflect the true assay range, control and background data are explicitly included in the fitting. Blank samples (background) are incorporated at a high nominal concentration (10^10^ μg/mL) to anchor the Bottom asymptote, thereby incorporating background noise into the model estimation. Control samples are assigned a dose of 0 μg/mL to define the Top asymptote.

### 2.10. Normalization and IC_50_ Determination

Once the Top and Bottom parameters are obtained from the raw data fit, all raw data points are transformed into a percentage of cell viability using the normalization equation (cf. Equation (5)) above. From the normalized curve, the IC_50_ is obtained as the drug concentration corresponding to 50% inhibition.

This two-step approach provides a robust parameter estimation from raw data while normalizing curves that allow for direct comparison of DRC across multiple experiments.

### 2.11. Confocal Microscopy in PC3 Cancer Cells

PC3 cells were prepared in Petri dishes to be observed under confocal microscopy. The cells are first extracted through the process of passage. A total of 15 μL of Trypan Blue solution was added to 15 μL of the resuspended pellet in RPMI medium. This solution was used to calculate the dilution needed to prepare the desired amount of solution with a concentration of 7 × 10^4^ live cells/mL. After adding the amount of 7 × 10^4^ live cells/mL, a solution with the appropriate working volume of the treated cells was incubated for 24 h. Following the incubation period, the cells are washed and prepared with PBS before adding the working volume of the glycerol (50%) mounting medium, which is the final step to prepare the cells for confocal microscopy analysis.

### 2.12. Morphological Studies in Human Red Blood Cells

Human blood was obtained from healthy volunteers from men aged between 25 and 35 years, and collected in heparinized tubes (Terumo Medical Co., Elkton, MD, USA) to prevent coagulation. Erythrocytes were isolated by centrifugation at 4000× *g* for 5 min at 5 °C using a refrigerated Beckman-52 centrifuge. The buffy coat and plasma were carefully removed by aspiration. Red blood cells were subsequently washed three times with ice-cold isotonic buffer (pH 7.4) composed of 5 mM KCl, 10 mM Tris or cacodylic acid, 5 mM glucose, and sufficient NaCl to reach a final ionic strength of 155 mM. After the final wash, cells were resuspended in the same buffer to a final 30% hematocrit (HCT). HCT was adjusted using a Clay–Adams Readacrit centrifuge from Becton Dickinson (BD; Parsippany, NJ, USA). Next, 0.4 mL of the diluted RBC suspension was combined with 1.2 mL of nanocomposite dispersions in PBS at final concentrations of 1.75, 8.76, and 17.9 µg/mL for both gold nanocomposites and ADG-loaded gold nanocomposites. The mixtures were gently homogenized using a roller mixer and incubated at room temperature for 3 h. Red blood cell deformability and aggregation were assessed at hourly intervals throughout the incubation period.

For the RBC morphological assay, aliquots collected at different time points were diluted to a final hematocrit of 2.5–5% and fixed with 250 µL of 0.025% glutaraldehyde. The fixed cells were then loaded into a hemocytometer and examined microscopically. Cell morphology was assessed and manually counted using an Olympus CX33 microscope equipped with an Olympus EP50 camera manufactured by Evident Corporation (Tokyo, Japan).

### 2.13. Red Blood Cell Hemolysis Assay

Human blood hemolysis assays were conducted using aliquots of the same RBC samples, exposed to gold nanocomposites and ADG-loaded gold nanocomposites at concentrations of 1.75, 8.76, and 17.9 µg/mL, following a 3 h 37 °C incubation period. The samples were then centrifuged at 12,000× *g* for 30 s, and a 200 µL aliquot of the supernatant was collected. Hemoglobin release was quantified by measuring absorbance at 540 nm using a spectrophotometer (Synergy™ HT, BioTek^®^, Winooski, VT, USA). The absorbance values were normalized against those obtained from completely hemolyzed cells, which served as the 100% lysis control and the percentage of hemolysis was calculated relative to the control cells. Hemolysis rate (%) was calculated using a standard formula:

(6) $$\left(\frac{Samples\;\left(abs\right)}{Total\;hemolysis\;\left(abs\right)}\right)\;\times \;100\%$$

### 2.14. Instrumentation

Powder diffractograms (PXRDs) were collected in transmission mode (100 K) using a Rigaku XtaLAB SuperNova X-ray diffractometer (Wrocław, Poland) with a micro-focus Cu-Kα radiation (λ = 1.5417 Å) source and equipped with a HyPix3000 X-ray detector (50 kV, 0.8 mA). Powder samples were mounted in MiTeGen micro loops. Powder diffractograms were collected between 6 and 60° with a step of 0.01° using the Fast phi move experiment with 300 s of exposure time. NMR spectra were obtained using a Bruker Ascend Aeon 500 MHz (Billerica, MA, USA) equipped with multinuclear capabilities, variable temperature settings, and cross-polarization magnetic angle spinning. The samples were analyzed at room temperature, using D_2_O and dimethyl sulfoxide (DMSO) as solvents. The solvent signals at 4.80–4.81 and 2.50 ppm were used as internal standards for protons. Raman spectra were recorded in a Thermo Scientific DXR Raman microscope (Waltham, MA, USA), equipped with a 532 nm laser, 400 lines/nm grating, and 50 μm slit. The spectra were collected at room temperature over the range of 3,400 to 100 cm^−1^ by averaging 32 scans with exposures of 5 s. The OMNIC for Dispersive Raman software version 9.2.0 was employed for data collection and analysis. UV–Vis absorption spectra were acquired using a Thermo Scientific Genesys 10S UV–Vis spectrophotometer (Waltham, MA, USA). Fourier transform infrared (FTIR) spectra were captured employing a Bruker Optik GmbH Tensor 27 FTIR system (Ettlingen, Germany). For the examination of the morphology and dimensions of the nanocomposites, X-ray microanalysis was conducted utilizing a JEOL JSM-6480LV scanning electron microscope (Peabody, MA, USA) equipped with an Everhart–Thomson secondary electron imaging (SEI) detector and an energy dispersive X-ray analysis (EDS) Genesis 2000 detector. Additional analyses were performed with an FEI TALOS 200x high-resolution scanning/transmission electron microscope (TEM; Peabody, MA, USA). Confocal microscopy was done with Nikon Ti Microscope from Nikon USA (Melville, NY, USA) with a S Fluor 40× Oil DIC H N2 objective. Confocal microscopy channels, Alexa Fluor 488 (Exc 487.5 nm, Em 525.0 nm) and GFAP CY3 (Exc 561.5 nm, Em 595.0 nm), were used with a Laser Scan Confocal GaAsP modality and TD (Exc N/A, Em N/A) with a Laser Scan Confocal TD modality. Capturing was done with a Z step of 0.3 μm with a count of twenty-five.

## 3. Results

### 3.1. Synthesis and Characterization of Gold–Carbon Quantum Dots and Doped Derivatives

**Figure 1:**
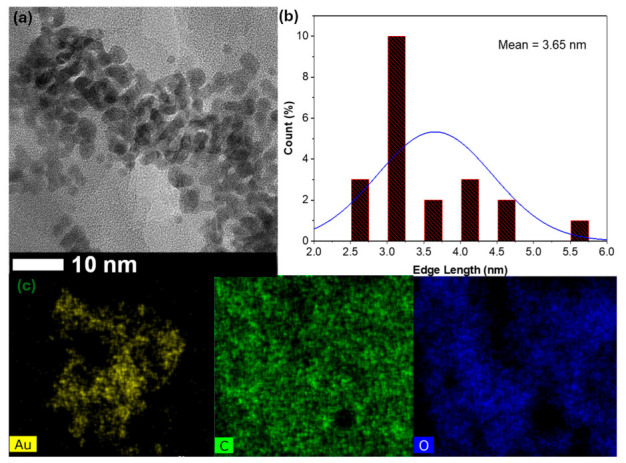
Characterization of AuCBQD nanoparticles: (**a**) HR-TEM image of AuCBQD particles, (**b**) size distribution (histogram) of AuCBQD particles, and (**c**) EDS element mapping of Au, C, and O within the particles.

The morphology of AuCBQD was characterized by HR-TEM (Figure 1a), showing spherical and monodisperse particles. The histogram analysis yielded an average diameter (edge length) of 3.65 ± 0.1 nm (Figure 1b). HAADF-EDS mapping confirmed the presence of Au, C, and O (Figure 1c). AuNCBQD exhibited dispersed spherical particles (Figure S1a) with an average diameter of 5.79 ± 0.1 nm (Figure S1b) and elemental composition including Au, C, O, and N (Figure S1c). AuSCBQD displayed non-uniform morphologies (Figure S2a) and a broad size distribution with an average diameter of 6.16 ± 0.1 nm (Figure S2b); HAADF-EDS mapping confirmed Au, C, O, and S (Figure S2c) (Figures S1 and S2). EDS mapping of AuCBQD and AuSCBQD is shown in Figure S3a and S3b, respectively.

**Figure 2:**
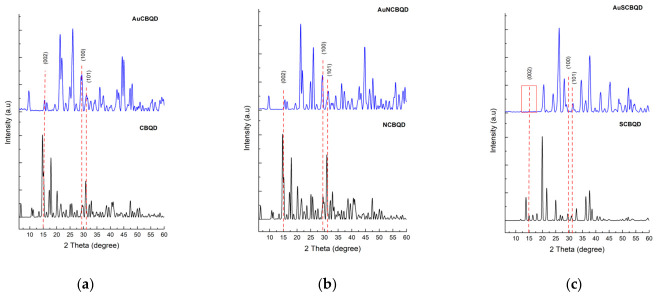
XRD pattern comparisons of carbon-based nanoparticles with and without gold from bottom to top: (**a**) CBQD and AuCBQD, (**b**) NCBQDs and AuNCBQD, and (**c**) SCBQD and AuSCBQD.

The crystallinity of the synthesized gold nanocomposites was examined by XRD. Figure 2 compares CBQDs, D-CBQDs, and the synthesized gold nanocomposites. All gold nanocomposites exhibited a distinct Bragg reflection at 2θ = 44.5°, which could be attributed to the (200) plane of face-centered cubic gold (Figure 2a–c). However, the peak at ~38° (corresponding to Miller indices (111), which should be the most intense is not observed. Graphite-related planes (002), (100), and (101) were observed, and peak displacements relative to CBQD were detected for AuCBQD and AuNCBQD (Figure 2a,b). In AuSCBQD, the CBQD (002) signal at ~15° was not observed (Figure 2c).

UV–Vis spectra comparing CBQD/NCBQD/SCBQD with their corresponding gold nanocomposites are shown in Figure S4. CBQD exhibited a band at 254 nm (Figure S4a). NCBQD exhibited two bands at 254 and 330 nm (Figure S4b). SCBQD exhibited an absorption band at 254 nm (Figure S4c). The synthesized Au nanocomposites lacked the characteristic LSPR band typical of bare AuNPs. FT-IR spectra for CBQDs/D-CBQDs and corresponding gold nanocomposites are shown in Figure S5. Broad absorption bands in the 3100–3500 cm^−1^ region were detected, corresponding to the stretching vibrations of O–H bonds. Additional peaks appeared at approximately 1563, 1628, and 1384 cm^−1^, which are likely from the bending vibration of the C=C bond of CBQDs. The characteristic carboxyl C=O stretching bands are observed around 1635 and 1715 cm^−1^. Peaks at 1285, 1215, and 894 cm^−1^ correspond to the C–N and C–S stretching modes in NCBQD and SCBQD, respectively, and analogous features were observed after gold nanocomposite formation.

**Figure 3:**
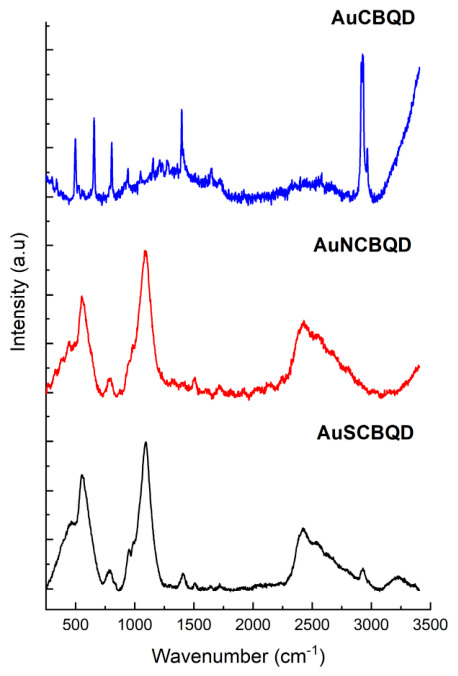
Raman spectra comparison of AuCBQD (black), AuSCBQD (red), and AuNCBQD (blue).

Raman spectroscopy was employed to explore the interactivity between the AuNPs, CBQD, and D-CBQDs. Representative D and G bands located at 1348 and 1595 cm^−1^ were observed (Figure 3) and shifts relative to the carbon quantum dot precursors were detected.

### 3.2. Synthesis and Characterization of Andrographolide-Coated Gold–Carbon Quantum Dots and Their Doped Derivatives

**Figure 4:**
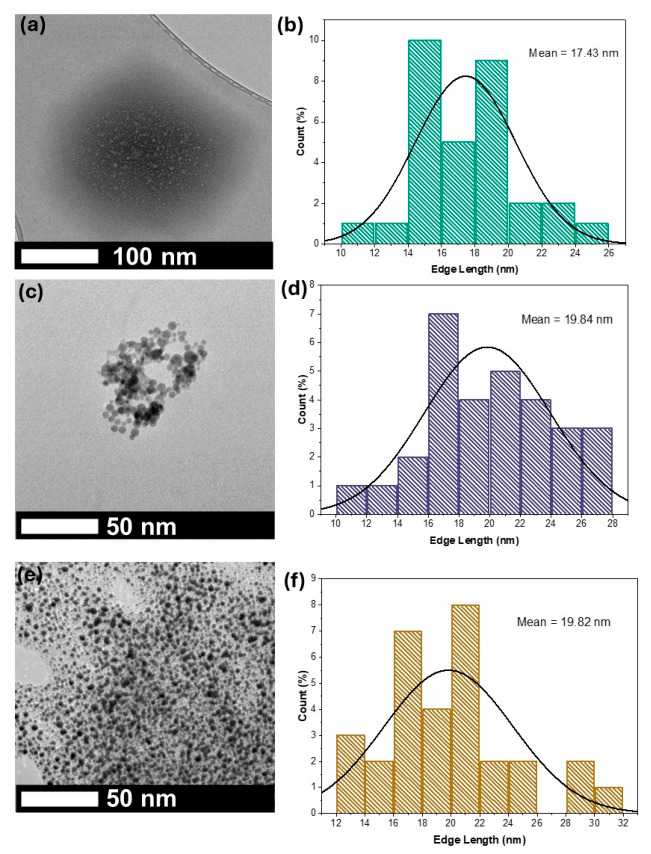
HR-TEM images of (**a**) AuCBQDADG, (**c**) AuNCBQDADG, (**e**) AuSCBQDADG, and particle size distributions of (**b**) AuCBQDADG, (**d**) AuNCBQDADG, and (**f**) AuSCBQDADG.

**Figure 5:**
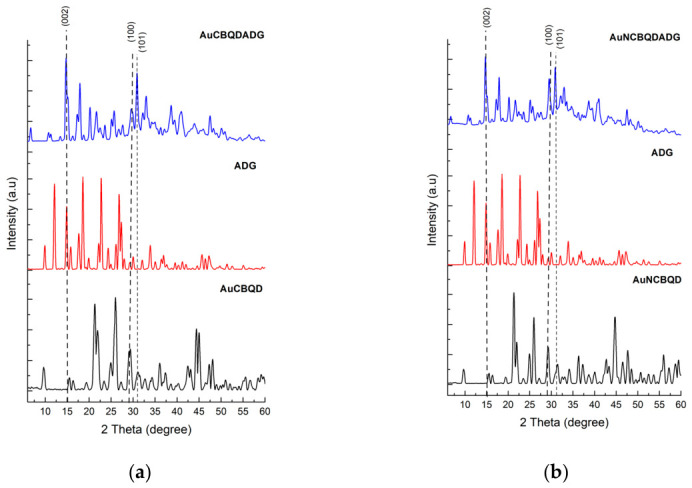
XRD pattern comparison of (**a**) AuCBQD, ADG, and AuCBQDADG, and (**b**) AuNCBQD, ADG, and AuNCBQDADG.

TEM images of ADG-loaded nanocomposites are shown in Figure 4. AuCBQDADG exhibited spherical particles (Figure 4a) with an average diameter (edge length) of 17.43 ± 0.2 nm (Figure 4b). AuNCBQDADG displayed spherical particles (Figure 4c) with an average diameter of 19.84 ± 0.3 nm (Figure 4d). AuSCBQDADG exhibited spherical particles (Figure 4e) with an average diameter of 19.82 ± 0.6 nm (Figure 4f). XRD patterns for ADG, gold nanocomposites, and ADG-loaded gold nanocomposites are shown in Figure 5. Similarly, for uncharged gold nanocomposites, the peak at 44.5° was observed in all loaded gold nanocomposites. In ADG-loaded nanocomposites, the characteristic graphite planes (002), (100), (101), and (012) at 15° of ADG were observed (Figure 5a,b).

**Figure 6:**
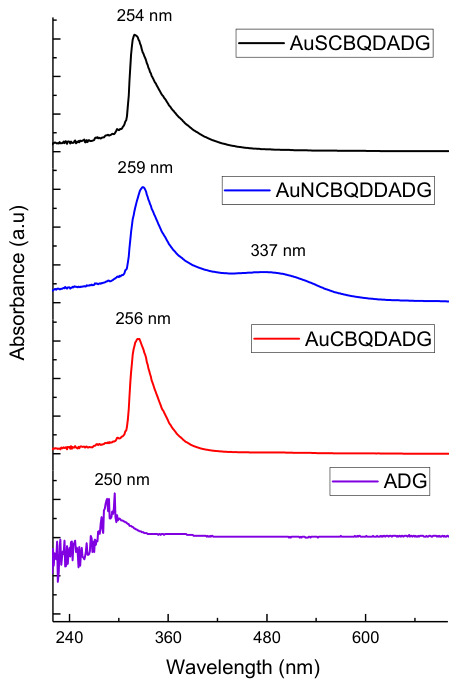
UV–Vis spectra comparison from bottom to top: ADG, AuCBQDADG, AuNCBQDADG, and AuSCBQDADG.

UV–Vis spectra of ADG and loaded nanocomposites are shown in Figure 6. ADG exhibited a characteristic absorption peak at ~250 nm (Figure 6). After loading, absorption maxima were observed at ~256 nm (AuCBQDADG), ~259 nm (AuNCBQDADG), and ~254 nm (AuSCBQDADG). AuNCBQD also shows a band at 337 nm (Figure 6).

FT-IR spectra comparing AuCBQD, AuNCBQD, AuSCBQD, ADG, and ADG-loaded materials are shown in Figure S6. In ADG-loaded nanocomposites, peaks at 3500, 2976, 2890, 1645, 1079, 1043, and 875 cm^−1^ likely corresponding to the O-H, C-H, C=O, C=C stretching, O-H for primary and the secondary alcohol features, and C-O-C in the lactone ring (which is present in the molecular structure of ADG), respectively, were observed.

### 3.3. NMR Studies

^1^H NMR spectra comparing CBQD, D-CBQDs, and synthesized gold nanocomposites are shown in Figure S7. In CBQDs, signals between 2.31 and 2.45 ppm are attributed to allylic protons, while peaks at 5.25–5.75 ppm correspond to vinylic protons. An additional signal at 3.03–3.24 ppm is assigned to hydroxyl (O–H) protons. Comparable spectral features were observed for AuCBQD (Figure S7a). In contrast, AuNCBQD exhibited additional peaks and altered splitting patterns compared to NCBQD, including a new signal at approximately 6.52 ppm and another at 2.17 ppm (Figure S7b). AuSCBQD also showed chemical shift variations relative to SCBQD (Figure S7c), indicating a structural modification following gold incorporation.

**Figure 7:**
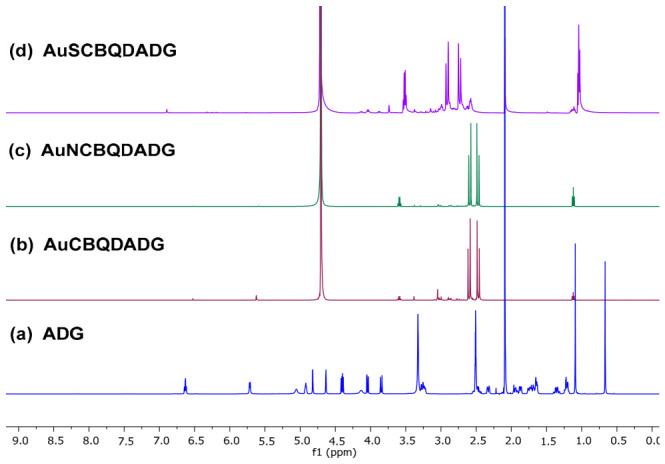
^1^H NMR spectra of the different samples from bottom to top: (**a**) ADG, (**b**) AuCBQDADG, (**c**) AuNCBQDADG, and (**d**) AuSCBQDADG.

^1^H NMR spectra of ADG and ADG-loaded nanocomposites are shown in Figure 7. For AuCBQDADG (Figure 7a), the characteristic ADG aliphatic proton signals (0.65–0.99 ppm) disappeared. Additionally, spectral changes were observed at 6.61–6.64 ppm and 5.70–5.72 ppm, corresponding to vinylic protons from ADG and AuCBQD, respectively. New signals emerged at 1.11–1.13 ppm, suggesting the presence of modified aliphatic environments. In AuNCBQDADG (Figure 7b), the disappearance of ADG-associated signals at 0.56, 0.99, and 1.10–1.98 ppm, associated with the vinylic protons present, was observed. Chemical shift changes occurred at 3.43, 3.69, 4.13, and 5.64 ppm, accompanied by new signals at 1.10–1.13 and 3.61 ppm, further supporting successful incorporation. Similarly, AuSCBQDADG (Figure 7c), displayed new vinylic proton signals at 6.26, 6.73, and 6.89 ppm, along with noticeable shifts at 2.85 and 5.77 ppm. Overall, the spectral changes confirm successful gold incorporation and ADG loading, as evidenced by peak shifts, signal disappearance, and the appearance of new resonances.

### 3.4. Partition Coefficients Results and General Solubility Equation (GSE) Studies

Partition coefficient results determined by the NMR method are summarized in Table 1 (unloaded gold nanocomposites) and Table 2 (loaded gold nanocomposites). CBQD and AuCBQD showed log P values of −0.193 ± 0.026 and −0.152 ± 0.006, respectively. The log P values for NCBQD and AuNCBQD were observed to be 0.759 ± 0.158 and 1.008 ± 0.027, respectively. SCBQD exhibited log P values of −0.614 ± 0.104, while AuSCBQD was found to have a value of −0.276 ± 0.024 (Table 1). These findings align with previous studies reported [36].

In all instances where ADG was coupled with gold nanocomposites, an improvement in drug solubility was observed (Table 2). Free ADG exhibited a log P of 1.106 ± 0.018. while AuCBQDADG, AuNCBQDADG, and AuSCBQDADG were found to have measured values of −0.104 ± 0.006, 0.123 ± 0.002, and −0.095 ± 0.005, respectively. Notably, gold nanocomposites doped with sulfur and coupled with ADG showed the highest degree of hydrophilicity.

GSE calculation outputs based on the data in Table 1 and Table 2 are summarized in Table 3. The results highlight a significant increase in relative water solubility: 546-fold for ADG with AuCBQD, 322-fold with AuSCBQD, and 534-fold with AuNCBQD.

### 3.5. Cytotoxicity Assays

**Figure 8:**
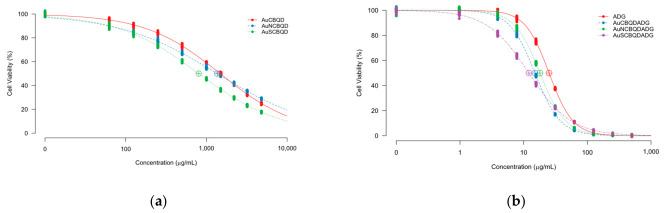
IC_50_ curves for (**a**) AuCBQD (red line), AuNCBQQD (blue line), AuSCBQQD (green line), and (**b**) ADG (red line), AuCBQDADG (blue line), AuNCBQDADG (green line), and AuSCBQDADG (violet line) using PC3 cell line at 48 h of treatment.

Cytotoxicity in PC3 cells was evaluated after 48 h treatment using dose–response curves (Figure 8) and IC_50_ values (Table 4). Unloaded nanocomposites were tested at concentrations ranging from 0 to 5000 µg/mL and yielded IC_50_ values of 1474 ± 5 µg/mL (AuCBQD), 1357 ± 5.6 µg/mL (AuNCBQD), and 8.02 ± 5.6 µg/mL (AuSCBQD) (Figure 8a; Table 4). ADG and ADG-loaded nanocomposites were tested at concentrations ranging from 0 to 500 µg/mL.

ADG exhibited an IC_50_ value of 25.05 ± 0.06 µg/mL. In comparison, AuCBQDADG showed a lower IC_50_ of 15.03 ± 0.04 µg/mL, AuNCBQDADG demonstrated an IC_50_ of 18.00 ± 0.04 µg/mL, and AuSCBQDADG displayed the lowest IC_50_ value at 12.08 ± 0.04 µg/mL (Figure 8b; Table 4). A lack-of-fit test was performed to assess the model’s ability to represent the experimental results adequately (Table S1). In addition, the performance of the four-parameter log-logistic (LL.4) models was compared with linear regression models using the Akaike Information Criterion (AIC) (Table S2). A p-value greater than 0.05 indicated an acceptable fit. The results provide strong statistical evidence that the dose–response curves provide the most accurate fit to the experimental data.

### 3.6. Confocal Microscopy

**Figure 9:**
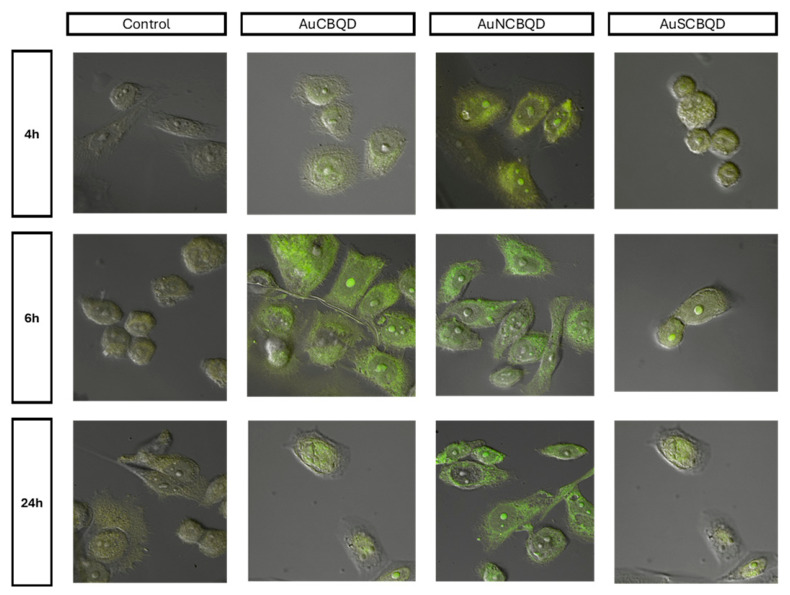
Confocal microscopy of AuCBQD, AuNCBQD, and AuSCBQD incubated with PC-3 cells at timeframes of 4, 6, and 24 h. Deconvolution was done via NIS-Elements Offline Deconvolution software (Version 5.41.00).

Confocal microscopy was performed to assess the cellular uptake of AuCBQD, AuNCBQD, and AuSCBQD in PC3 cells compared to the decrease in intensity of the control (single cells) at 4, 6, and 24 h (Figure 9). At 4 h, AuCBQD, AuNCBQD, and AuSCBQD were observed within PC3 cells and localized predominantly in the perinuclear region. At 6 h and 24 h, nanocomposites remained localized in vesicular structures surrounding the nucleus, with no evidence of cytoplasmic diffusion. Intensity confocal microscopy was performed to compare the decrease in intensity of the control (single cells) when exposed to gold–carbon quantum dots nanocomposites and their derivatives in PC3 cell lines (Figure S8). For all three materials (AuCBQD, AuNCBQD, AuSCBQD), an increase in fluorescence was observed at 4 and 24 h. Specifically, AuCBQD and AuSCBQD exhibited higher intensity at 6 h, while AuNCBQD showed higher intensity at 24 h.

### 3.7. Morphological Studies in Human Red Blood Cells (RBCs)

RBC morphology exposure to gold nanocomposites at 1.75, 8.76, and 17.9 µg/mL was evaluated, and potential hemolysis was assessed by measuring hemoglobin (Hb) absorbance at 540 nm. The results are summarized in Table S3 and illustrated in Figures S9 and S10. Table S3 presents the percentages of discocytes, stomatocytes, echinocytes, and dumbbell-shaped cells for AuCBQD, AuNCBQD, and AuSCBQD at each concentration, together with the corresponding hemolysis values. Overall, stomatocytes were the predominant morphological form, accounting for 80–99% of the observed alterations. For AuCBQD, the percentage of discocytes was 8.13%, 7.80%, and 7.97% at concentrations a–c, respectively, while stomatocytes accounted for 86.33%, 84.73%, and 90.06%. Hemolysis values were 3.02%, 6.73%, and 6.09%. For AuNCBQD, discocytes were observed at 0%, 0%, and 1% across concentrations a–c, whereas stomatocytes represented 98.87%, 99.46%, and 98.12%. The corresponding hemolysis values were 6.09%, 5.83%, and 11.69%. For AuSCBQD, discocytes accounted for 6.02%, 6.65%, and 6.46%, while stomatocytes comprised 92.50%, 92.06%, and 92.61% at concentrations a–c, respectively. Hemolysis percentages were 8.24%, 3.10%, and 2.26%. Representative microscopic images comparing control RBCs with cells exposed to AuCBQD, AuNCBQD, and AuSCBQD at concentrations a–c are shown in Figure S9. Table 5 summarizes the effects of ADG and ADG-loaded gold nanocomposites on red blood cells (RBCs). RBCs in the control group preserved their characteristic discocyte morphology. In contrast, exposure to three different concentrations of ADG-loaded gold nanocomposites induced marked morphological alterations, with cells adopting stomatocytic, dumbbell-shaped, and echinocytic forms. These changes are consistent with those previously reported for gold nanocomposites (Figure S10). Notably, among the tested materials, only AuCBQDADG at concentrations of 8.76 and 17.9 µg/mL produced hemolysis values exceeding 5%, although remaining below 10%. In comparison, all other ADG-loaded gold nanocomposites yielded hemolysis percentages below 5%, classifying them within the non-hemolytic to slightly hemolytic range.

## 4. Discussion

This study demonstrates a one-step strategy for synthesizing gold–carbon quantum dot (Au–CBQD) nanocomposites and their doped derivatives, followed by successful conjugation with ADG. The combined structural, spectroscopic, physicochemical, and biological analyses confirm the formation of stable nanocomposites and their association with ADG without altering the crystalline structure of the gold core. CBQDs and D-CBQDs functioned as both reducing agents for Au^3+^ and stabilizing shells surrounding the resulting gold nanoparticles.

XRD analysis confirmed the characteristic graphite planes (002), (100), and (101). However, the AuCBQD and AuNCBQD showed a displacement in the 3 main peaks of the CBQD (002, 100, and 101), consistent with an interaction between the gold and the carbon nanoparticles, along with the absence of the (002) reflection in AuSCBQD. This suggests the formation of a new type of interaction, which can be explained by the binding of SCBQDs to AuNPs via Au-thiol interactions. Gold nanoparticles displayed four characteristic diffraction peaks at 2θ values of 38.1°, 44.3°, 64.5°, and 77.7°. These peaks are attributed to the standard Bragg reflections from the (111), (200), (220), and (311) planes of a face-centered cubic (fcc) lattice [45,46]. Although a peak at 2θ = 44.5 was observed in all synthesized gold-nanocomposites, it cannot be definitively assigned to the (200) plane of face-centered cubic gold, as might initially be assumed. This is because the peak is relatively small and sharp. Moreover, the absence of characteristic FCC gold peaks (111 and 200) suggests that gold is not present in the form of bulk metallic particles. Instead, it is likely highly dispersed or exists as clusters smaller than 5 nanometers within the CBQDs and D-CBQDs framework. At this scale, the particle size may fall below the detection limit of XRD, or the diffraction peaks may be so broadened that they merge into the background [47]. Furthermore, when ADG is coupled with the gold nanocomposites, it likely forms polar functional groups that interact with ADG’s functional groups through electrostatic interactions, hydrogen bonding, and/or π−π stacking interactions. These interactions increase the adsorption energy of the drug on the carrier surface [48]. In the cases of AuCBQDADG and AuNCBQDDADG, some characteristic signals of ADG are suppressed [(002), (100) at 10°, 11.9°, respectively], likely due to the arrangement of ADG within the nanocomposites.

Raman spectra show the two representative peaks located at 1348 and 1595 cm^−1^, which can be assigned as D and G bands arising from the presence of defect sites in the graphite and tangential C−C bond vibrations [49,50], and suffered a shift at 556 and 1092 cm^−1^. This could result from a decrease in the quantity of unsaturated carbon sites, attributed to the bonding of Au to these sites through the formation of Au-C bonds. On the other hand, the intensity ratio is an indicator of the degree of disorder or doping: a higher value means a higher degree of doping and lattice defects; therefore, the relative intensity of the D peak depends on the atomic size difference between the dopant and carbon. These results suggest that the CBQD and D-CBQDs wrapped on AuNPs lead to the synthesized gold nanocomposites displaying a strong Raman signal.

Gold nanoparticles and CBQDs exhibit unique plasmonic and photonic characteristics [51]. CBQD exhibits a strong band at 254 nm consistent with the uniform size of sp2 clusters, NCBQD shows two absorption bands at 254 and 330 nm; the latter can be attributed to the n−π* transition of the C−N, whereas SCBQD also exhibits an absorption band at 254 nm, which is attributed to the doping of sulfur element along with the strong absorption band due to the graphitic structure. Gold nanoparticles exhibit an absorbance band in the visible region between 500 and 600 nm, depending on the size of the particles [52]. Notably, the synthesized gold nanocomposites did not exhibit the characteristic localized surface plasmon resonance (LSPR) band typically observed in citrate-reduced AuNPs. This observation aligns with substantial surface coverage by CBQDs and D-CBQDs, both of which can significantly influence optical absorption properties, as previously reported [53]. Furthermore, the presence of a carbon dot (CD) coating may suppress the characteristic plasmon resonance peak of AuNPs through mechanisms such as surface passivation and charge transfer [54,55]. Nocito et al. reported the photochemical synthesis of CD-Au nanohybrid in which CDs act as reducing agents. Their findings indicate that the stability and optical properties of these hybrids are dependent on the size of the CDs and their capping effect, which can lead to suppressed or shifted plasmonic signals depending on the synthesis conditions [56]. Similarly, Zhao et al. explored the synergistic interactions between CDs and Au nanoparticles. Although their work focuses on photo-catalytic enhancement, they observed that electronic coupling between the two components alters LSPR characteristics and facilitates hot carrier injection, thereby modifying the resulting absorption spectra [57]. Based on this, it is worth mentioning that all gold nanocomposites underwent a red shift, consistent with the formation of gold–carbon nanoparticles. On the other hand, ADG exhibits a characteristic peak at ~250 nm, attributed to the π → π* electronic transition of its two olefin bonds [58]. These peak shifts to around 256, 259, and 254 nm for AuCBQDADG, AuNCBQDADG, and AuSCBQDADG, respectively, upon the conjugation with the nanocomposites. AuNCBQD also shows a band at 337 nm, which can be attributed to the n−π* transition of the C−N [59]. This shift in the absorption peak is likely due to π−π stacking and hydrophobic interactions between ADG and the abundant aromatic rings in CBQDs surrounding the gold nanoparticles, as reported [17]. Additionally, it should be noted that the absorption peak of ADG is similar to those of the gold–carbon-based quantum dot nanocomposites, which could result in peak overlap.

A central finding of this work is the marked shift in hydrophobicity following ADG conjugation, and to elucidate the surface chemistry of ligand shells on AuNPs, NMR is an effective tool [60,61,62]. In ^1^H NMR spectra, an alternate splitting pattern was observed around 6.52 ppm, likely attributable to the presence of a pyrrole group, a characteristic feature often observed in NCBQDs [36,63,64]. The minimal disparity in electronegativity between S (2.58) and C (2.55) is very small, implying a limited driving force for electron transfer between them [63]. As a consequence, by comparing SCBQD and AuSCBQD NMR spectra, a downfield shift was observed due to the C atom being slightly less electronegative than S, creating a deshielding effect in the coating of gold nanoparticles [65]. The alteration in splitting patterns, the emergence of new signals, and the vanishing of specific signals serve as indications of the interaction occurring between the gold nanoparticles and CBQDs and D-CBQDs. The presence of AuCBQD and Au-DCBQD signals, along with ADG signals in the AuCBQDADG and AuD-CBQDADG ^1^H NMR spectra, indicates the successful loading of ADG onto the quantum dots. Changes in splitting patterns, the appearance of new signals, and the disappearance of certain signals provide evidence of the coupling interaction between ADG and the AuCBQDs and Au-DCBQDs. The hydrophobic/hydrophilic character is a key parameter for a nano-carrier [66,67]. A compound that is more polar and hydrophilic tends to exhibit a lower log P value, possibly even dipping into the negative range, indicating a preference for the aqueous phase. Conversely, compounds that are more non-polar and hydrophobic will show a higher log P, leading them to partition into the organic phase [68]. CBQD displays hydrophilic traits owing to the presence of oxygen-containing functional groups on its surface, which enhance its solubility in water [69]. Additionally, it is noteworthy that AuCBQD demonstrates superior hydrophilicity when compared to AuNCBQD and AuSCBQD. This discrepancy can be attributed to the hydrophobic nature of nitrogen atoms, as they are relatively nonpolar, while sulfur exhibits hydrophilic properties compared to nitrogen. These findings align with previous studies conducted regarding CBQD and D-CBQD [36]. Free ADG exhibited a log P of 1.106, whereas AuCBQDADG and AuSCBQDADG exhibited negative log P values, and AuNCBQDADG exhibited a near-neutral value. In all instances where ADG was coupled with gold nanocomposites, an improvement in drug solubility was observed.

Determining the lipophilicity of nanocarriers containing ADG has become increasingly complex compared to measuring the drug alone. The incorporation of ADG into nanocarriers can alter its apparent lipophilicity. For instance, certain nanocomposites have been reported to reduce the log P value of ADG from 2.63 (highly lipophilic) down to 0.56 (hydrophilic) [70]. High-performance liquid chromatography (HPLC) provides an indirect method for estimating log P, where retention time on a C18 column is compared with reference standards of known lipophilicity [71]. An example is the study by Liu et al., who evaluated the log P of ADG nanocrystals modified with a newly synthesized sodium dodecyl sulfate (SDS)- d-α-Tocopherol polyethylene glycol 1000 succinate (TPGS) copolymer. Measurements were conducted in purified water at pH 1.2, pH 4.0, and pH 6.8 at 25 °C and 37 °C using HPLC. The result shows that the log P values of these nanocarriers decreased to a range of 1.6–1.1, indicating increased hydrophilicity compared to unmodified nanoparticles. Notably, the modified nanoparticles exhibited greater hydrophilicity at 37 °C than at 25 °C [72]. However, HPLC-based methods cannot distinguish whether changes in log P arise from the encapsulation of the drug within the carrier or from chemical modifications at the carrier’s surface. NMR spectroscopy, in contrast, can provide more reliable insight into the origin of these changes [73]. This capability was a key reason for its use in the present study for log P determination.

GSE is a dependable approach for estimating the molar aqueous solubility (Sw) of nonelectrolytes [74], providing insights into potential solubility trends. AuCBQDADG and AuSCBQDADG exceeding 12,000 µg/mL. These values represent several-hundred-fold increases compared to ADG’s Sw using its log P from literature for the GSE [75]. Previous studies [76] have reported improvement in ADG solubility using nanoemulsion-based systems; however, the present strategy achieves enhanced solubility while maintaining ADG in an aqueous nanocomposite formulation.

Cytotoxicity studies in PC3 prostate cancer cells demonstrated that ADG-loaded nanocomposites retained anticancer activity. The PC3 cell line, androgen-independent grade IV adenocarcinoma, produces prostate-specific antigen (PSA) but does not express an androgen receptor (AR) [77,78]. Among unloaded nanocomposites, AuSCBQD exhibited significantly lower IC_50_ compared to AuCBQD and AuNCBQD, indicating that sulfur doping plays a key role in modulating biological activity. Sulfur forms stronger electronic interactions with the gold surface than nitrogen or carbon alone, leading to enhanced synergy within the hybrid structure. This effect improves the peroxidase-like or catalytic activity of the particles, increasing their ability to generate toxic species within the cell [79]. The primary mechanism for nanoparticle-induced cytotoxicity is the generation of Reactive Oxygen Species (ROS). While nitrogen doping primarily enhances fluorescence brightness, sulfur doping is often more effective at facilitating electron–hole separation, which directly translates to higher ROS production [80]. Elevated ROS levels can cause lipid peroxidation, DNA damage, and mitochondrial dysfunction, thereby lowering the IC_50_ [81]. These findings are consistent with published data on gold nanoparticles’ effects on various cancer cell lines [82].

The surface functionalization of gold nanoparticles plays a crucial role in their interaction with cells, influencing their potential effects and surface-chemical properties [83]. Surface functionalization and heteroatom incorporation are known to modulate nanoparticle–cell interactions, which may contribute to the observed differences. Free ADG exhibited an IC_50_ of 25.05 µg/mL [84], whereas AuCBQDADG, AuNCBQDADG, and AuSCBQDADG exhibited IC_50_ values of 15.03, 18, and 12.08 µg/mL, respectively.

Confocal microscopy demonstrated internalization of AuCBQD, AuNCBQD, and AuSCBQD into PC3 cells within 4 h, with persistent perinuclear vesicular localization at 6 and 24 h. Nanoparticle uptake is influenced by size, shape, surface coatings, and aggregation state [85,86]. Therefore, a deeper understanding of the interactions between AuNPs and biological membranes is necessary. Chan and coworkers demonstrated that AuNP uptake is highly size-dependent, with ~50 nm particles showing maximal uptake among 10–100 nm nanoparticles [87]. Surface charge has also been reported to influence cellular internalization. In the present study, all synthesized materials exhibited spherical morphology and were localized in vesicular structures without cytoplasmic diffusion. Such distribution is consistent with endocytic uptake mechanisms reported for gold nanoparticles, including macropinocytosis and receptor-mediated endocytosis [88,89]. Endocytosis is a process by which cells engulf substances for internalization, and it occurs in two main forms: macropinocytosis and receptor-mediated endocytosis.

Upon internalization by cells—primarily through endocytosis—the nanocomposites promote efficient intracellular delivery of the drug. Surface functionalization and heteroatom doping (e.g., sulfur or nitrogen), as demonstrated in this study, further enhance cellular uptake and interaction with biological membranes. Moreover, the intrinsic ROS-generating capability of the gold nanocomposites may act synergistically with ADG, amplifying oxidative stress and triggering apoptosis in cancer cells [90]. In addition, the nanocomposite platform may support controlled drug release, leading to increased intracellular accumulation of ADG at the target site.

Evaluation of red blood cell morphology and hemolysis provides additional insight into biocompatibility. Erythrocytes, commonly known as red blood cells (RBCs), constitute the majority of blood cells in humans and many other species. Their uncomplicated structure makes them highly accessible and relatively straightforward for engineers. Continuously generated in the bone marrow and replenished throughout life, RBCs remain in circulation for about 100–120 days [91]. Owing to this extended lifespan and steady renewal, they can serve as natural carriers for therapeutic agents and nanoparticles (NPs), enabling prolonged circulation while helping these substances evade the body’s clearance mechanisms [92]. Given the interest in employing gold nanocomposites for intravenous drug delivery, it is essential to examine how their interaction influences the unique morphology of RBCs. The characteristic biconcave shape and flexible membrane of RBCs have long served as a valuable model for investigating diverse physiological and metabolic processes [93,94,95]. RBC morphology assays were performed using different concentrations of compounds (1.75, 8.76, and 17.92 μg/mL) while monitoring for possible hemolysis by measuring the absorbance of hemoglobin (Hb) at 540 nm. Exposure to gold nanocomposites induced morphological transitions from discocytes to stomatocytes, echinocytes, and dumbbell-like forms, with stomatocytes representing the predominant morphology. These shape alterations highlight the remarkable deformability of RBCs, their capacity to undergo structural changes under external forces without rupturing. Such flexibility allows them to traverse capillaries narrower than their resting diameter [96].

Another key parameter to evaluate is hemolysis, the process in which RBCs rupture following damage to their membranes, resulting in the release of hemoglobin into the surrounding medium. While hemolysis is often associated with external stressors such as chemical agents or mechanical forces, it is also a natural event that occurs as RBCs reach the end of their lifespan. This physiological turnover ensures the continuous renewal of the blood cell population, but excessive or premature hemolysis can serve as an indicator of cytotoxicity or impaired membrane stability [97]. Evaluating hemolysis helps determine if the materials, during contact with RBCs, induce cell rupture. In erythrocyte hemolysis assays used to evaluate the biocompatibility of drugs or NPs, a hemolysis percentage of less than 5% is considered acceptable, as this range indicates that the material is safe and does not cause significant damage to red blood cells; values between 5% and 25% are classified as moderately hemolytic and reflect adverse effects that limit their biomedical use; While percentages above 25% are considered strongly hemolytic and therefore unacceptable for clinical applications according to international standards such as Biological evaluation of medical devices: Part 4: Selection of tests for interactions with blood ISO 10993-4 [98] and Standard Practice for Assessment of Hemolytic Properties of Materials (ASTMs)—F756-17 [99], both issued by the International Organization for Standardization. Among ADG-loaded materials, AuCBQDADG exhibited hemolysis values above 5% but below 10% at higher concentrations, while other formulations remained below 5%. In contrast, all other ADG-loaded gold nanocomposites produced hemolysis values under 5%, placing them within the non-hemolytic to slightly hemolytic range. Moreover, the presence of low hemolysis combined with distinct alterations in discocyte morphology strongly suggests that these materials interact predominantly with the red blood cell surface rather than penetrating or being internalized. Notably, the morphological shift from the typical biconcave discocyte shape to stomatocytes, characterized by a cup-like or concave form, indicates a significant modification of the cell membrane architecture. Such stomatocytic transformation is often associated with changes in membrane curvature and lipid bilayer asymmetry, reflecting surface-level interactions that perturb the outer leaflet of the membrane without causing extensive lysis. This observation reinforces the conclusion that the nanocomposites exert their effects through superficial contact with the erythrocyte membrane rather than by traversing or disrupting the intracellular environment. RBCs in the control group retained their characteristic discocyte morphology. In contrast, treatment with three distinct concentrations of ADG-loaded gold nanocomposites triggered pronounced morphological alterations, leading cells to assume stomatocytic, dumbbell-shaped, and echinocytic forms, changes consistent with those previously reported for gold nanocomposites.

The core material data shows that the native RBC discocytes experience minimal deformation compared to materials containing ADG, which exhibit pronounced deformation primarily towards stomatocytes, and notably, also towards echinocytes, unlike the response seen with gold nanocomposites. The cytoskeletal protein network of red blood cells endows them with a remarkable capacity to deform and accommodate external agents ranging from small molecules to macromolecules, while preserving membrane integrity and thereby conferring strong resistance to hemolysis [100,101]. These observations imply that the introduced materials interact directly with the RBC membrane, likely through ionic or hydrophobic associations, depending on the specific case. Based on the results obtained, a plausible mechanistic pathway for the incorporation of gold nanocomposites and ADG-loaded gold nanocomposites into red blood cells can be proposed. The evidence suggests that these nanocomposites exhibit strong affinity for outer-layer membrane proteins, favoring adsorption to the RBC surface while showing limited translocation across the bilayer. Such surface-dominated interactions are consistent with low hemolysis, indicating that the particles can circulate without causing irreversible membrane rupture. However, the observation of stomatocyte formation implies that beyond simple outer-leaflet binding, additional processes, such as perturbation of the inner leaflet, redistribution of membrane tension, or cytoskeletal coupling, may contribute to the curvature changes. Thus, while the nanocomposites primarily interact with membrane components rather than crossing through them, their influence on bilayer mechanics provides a plausible pathway for the shape transformations observed, reinforcing their potential for safe blood transport with subtle but reversible morphological effects.

Collectively, the data support that gold–carbon quantum dot nanocomposites and their doped derivatives provide a platform capable of enhancing ADG hydrophilicity while maintaining structural integrity, cellular uptake, cytotoxic activity, and acceptable hemocompatibility. The combination of one-step synthesis, tunable surface chemistry, and favorable physicochemical and biological characteristics establishes a foundation for further in vivo evaluation and development of nanocarrier-based strategies for anticancer drug delivery.

## 5. Conclusions

In this work, we produced gold nanocomposites using CBQDs, NCBQD, and SCBQD as reducing agents. UV–vis spectroscopy revealed that the presence of a carbon quantum dot shell did not suppress but rather modulated the absorbance peaks corresponding to gold nanoparticles. XRD analysis revealed that all gold nanocomposites exhibited the characteristic graphite planes (002), (100), and (101). Upon coupling ADG to the nanocomposites, several characteristic ADG signals were suppressed, likely due to the structural arrangement of ADG within the nanocomposites. FT-IR analysis confirmed the close bonding of CBQD and D-CBQD to the surface of AuNPs. ADG was coupled with synthesized gold nanocomposites based on carbon quantum dots and their derivatives, which exhibited a strong affinity for the aqueous phase. The nanocomposites were found to have spherical particles, and their size increased upon coupling with ADG due to the presence of the drug. NMR analysis was conducted to evaluate their partition coefficients, expressed as Log P. The log P of ADG decreased by approximately 3 logarithmic units when associated with these gold nanocomposites. Consequently, these nanosystems could enhance the transport and delivery of ADG in aqueous media, potentially leading to more effective therapeutic applications of ADG. Preliminary cytotoxicity evaluations of all materials indicated that both the unloaded and drug-loaded gold nanocomposites demonstrated substantial cytotoxic effects against PC3 cells, with greater activity compared to ADG (25.05 ± 0.06 µg/mL) after 48 h of exposure. However, it remains essential to perform cytotoxicity assessments of these nanocomposites in normal human prostate cells to ensure their safety and subsequently carry out in vivo experiments to validate the results presented in this work.

Confocal microscopy was employed to examine the cellular uptake of the gold nanocomposites into PC3 cell lines, indicating that the nanocomposites are internalized through endocytosis. It should be noted that, for a more comprehensive study, confocal imaging of the drug-loaded nanocomposites is also required. Morphological studies on human red blood cells (RBCs) exposed to three different concentrations of all materials showed that RBCs transformed into stomatocytes, dumbbells, and echinocytes, with hemolysis consistently below 5%, for the majority of the nanocomposites, suggesting no significant hemolysis occurred. This low hemolysis across all gold nanocomposites suggests their potential for safe transport through the bloodstream without causing irreversible damage. These findings demonstrate that these materials exhibit minimal cytotoxicity, positioning them as possible good DDS candidates capable of selectively targeting cancer cells.

## Figures and Tables

**Table 1:** Results of gold nanocomposites in terms of Log P Tests by the NMR Method.

Sample Name	Log P ($\bar{x}$)	SD
CBQD	−0.193	0.026
AuCBQD	−0.152	0.008
NCBQD	0.759	0.158
AuNCBQD	1.008	0.027
SCBQD	−0.614	0.104
AuSCBQD	−0.276	0.024

**Table 2:** Results of loaded-gold nanocomposites in terms of Log P Tests by the NMR method.

Sample Name	Log P ($\bar{x}$)	SD
ADG	1.106	0.018
AuCBQDADG	−0.104	0.006
AuNCBQDADG	0.123	0.002
AuSCBQDADG	−0.095	0.005

**Table 3:** Results for GSE calculations for gold-nanocomposites coupled with ADG.

Material	Sw (µg/mL)	Ratio (Sw Gold-Nanocomposites Coupled ADG/Sw ADG)
ADG	22.52 [44]	N/A
AuCBQDADG	12,289	545
AuNCBQDADG	7261	322
AuSCBQDADG	12,027	534

**Table 4:** IC_50_ of gold-nanocomposites and loaded-gold nanocomposites.

Material	IC_50_ (µg/mL)
AuCBQD	1474 ± 5
AuCBQDADG	15.03 ± 0.04
AuNCBQD	1357 ± 5.6
AuNCBQDADG	18.0 ± 0.04
AuSCBQD	802.02 ± 5.6
AuSCBQDADG	12.08 ± 0.04

**Table 5:** Predominant RCB morphologies after exposure to different concentrations (a = 1.75; b = 8.76; c = 17.9 µg/mL) of ADG-loaded-gold nanocomposites.

Materials at Different Concentrations (µg/mL)	Discocytes (%) 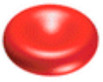	Stomatocytes (%) 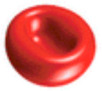	Echinocytes (%) 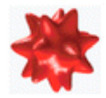	Dumbbells (%) 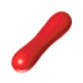	Hemolysis (%)
ADG ^a^	11.3	78.5	10.2	0	4.04
ADG ^b^	13.0	78.6	9.24	0	2.80
ADG ^c^	5.78	90.1	3.77	0	3.33
AuCBQDADG ^a^	11.9	85.6	2.03	0.37	2.70
AuCBQDADG ^b^	7.27	91.2	1.23	0.22	9.11
AuCBQDADG ^c^	6.11	92.3	0.82	6.90	6.12
AuNCBQDADG ^a^	10.9	85.4	3.40	0.15	4.18
AuNCBQDADG ^b^	8.49	90.1	0.93	0.44	1.77
AuNCBQDADG ^c^	8.80	88.4	1.86	0.90	1.86
AuSCBQDADG ^a^	10.4	85.8	2.89	0.12	3.39
AuSCBQDADG ^b^	8.56	92.6	1.83	0.37	2.90
AuSCBQDADG ^c^	9.05	91.7	0.98	0.24	2.41

## Data Availability

The original contributions presented in this study are included in the article/Supplementary Material. Further inquiries can be directed to the corresponding authors.

## Supplementary Materials

The following supporting information can be downloaded at https://www.mdpi.com/article/10.3390/pharmaceutics18060647/s1. Figure S1: Characterization of AuNCBQD nanoparticles: (a) HR-TEM image of AuNCBQD nanopar-ticles, (b) size distribution (histogram) of AuNCBQD nanoparticles, and (c) EDS mapping based on Au, C, O, and N within particles. Figure S2: Characterization of AuSCBQD nanoparticles: (a) HR-TEM image of AuSCBQD nanoparti-cles, (b) size distribution (histogram) of AuSCBQD nanoparticles, and (c) EDS mapping based on Au, C, O, and S within particles. Figure S3: EDS mapping of (a) AuCBQD particles, and (b) AuSCBQD particles. Figure S4: UV-Vis spectra comparison from bottom to top: (a) AuCBQD and CBQD, (b) AuNCBQD and N-CQBD, and (c) AuSCBQD and S-BQD. Figure S5: FT-IR spectra comparison from bottom to top: (a) CBQD and AuCBQD, (b) N-CQBD and AuNCBQD, and (c) S-CBQD and AuSCBQD. Figure S6: FT-IR spectra comparison from bottom to top: (a) AuCBQD, ADG, and AuCBQDADG, (b) AuNCBQD, ADG, and AuNCBQDADG, and (c) AuSCBQD, ADG, and AuSCBQDADG. Figure S7: ^1^H NMR spectra of the different samples from bottom to top: CBQD, D-CBQD, AuCBQD, AuNCBQD, and AuSCBQD. Figure S8: Intensity confocal comparison with the control (PC-3 cells without any material added) at different timeframes (4, 6, and 24 h) in (a) AuCBQD, (b) AuNCBQD, and (c) AuSCBQD. Figure S9: Microscopic images of the RBCs exposed to AuCBQD, AuNCBQD, and AuSCBQD at different concentrations (a = 1.75; b = 8.76; c = 17.9 µg/mL) compared to control (RBCs) at the same concentrations. Figure S10: Microscopic images of the RBCs exposed to AuCBQD, AuNCBQD, and AuSCBQD at different concentrations (a = 1.75; b = 8.76; c = 17.9 µg/mL) compared to ADG at the same con-centrations. Table S1: Lack-of-fit test performed for all results in cytotoxicity. Table S2: Model performance compared to linear regression using the Akaike Information Cri-terion (AIC). Table S3: Predominant RCBs morphologies after exposure to different concentrations (a = 1.75; b = 8.76; c = 17.9 µg/mL) of gold nanocomposites.

## Abbreviations

The following abbreviations are used in this manuscript:

CBQDs	Carbon-based quantum dots
D-CBQDs	Doped-CBQDs
AuNPs	Gold nanoparticles
LSPR	Localized surface plasmon resonance
ADG	Andrographolide
AuCBQDs	Gold–carbon quantum dots
AuD-CBQDs	Gold-doped carbon quantum dots
NMR	Nuclear magnetic resonance
RBCs	Human red blood cells
Log P	Partition coefficient
GSE	General solubility equation
S_w_	Molar aqueous solubility
AuCBQD	Gold nanoparticle covered with carbon-based quantum dots
AuNCBQD	Gold nanoparticle covered with nitrogen-doped carbon-based quantum dots
AuSCBQD	Gold nanoparticle covered with sulfur-doped carbon-based quantum dots
MTS assay	3-(4,5-dimethylthiazol-2-yl)-5-(3-carboxymethoxyphenyl)-2-(4-sulfophenyl)-2H-tetrazolium assay
IC_50_	Half-maximal inhibitory concentration
