# Supplementary material for: Andrographolide-Loaded Gold Carbon Quantum Dots and Their Doped Derivatives for Enhanced Hydrophilicity in a Drug Delivery System
Source: Pharmaceutics. 2026 May 24;18(6):647. doi: 10.3390/pharmaceutics18060647 (PMC13305392; doi:10.3390/pharmaceutics18060647)
Supplement: Supplementary file 1 [file pharmaceutics-18-00647-s001.zip › pharmaceutics-4234972-supplementary.pdf]

pharmaceutics

Supplementary Materials: Andrographolide-loaded gold carbon quantum dots and their doped derivatives for enhanced hydrophilicity in a drug delivery system

Wenndy Pantoja-Romero, Alexis Lavín Flores, Alejandro Lozada-Jerez, Mia Sara Perez-Salvá, Fabiola Rosa-Suárez, Orestes Quesada, Magaly Martínez-Ferrer, Gerardo Morell and Brad R. Weiner

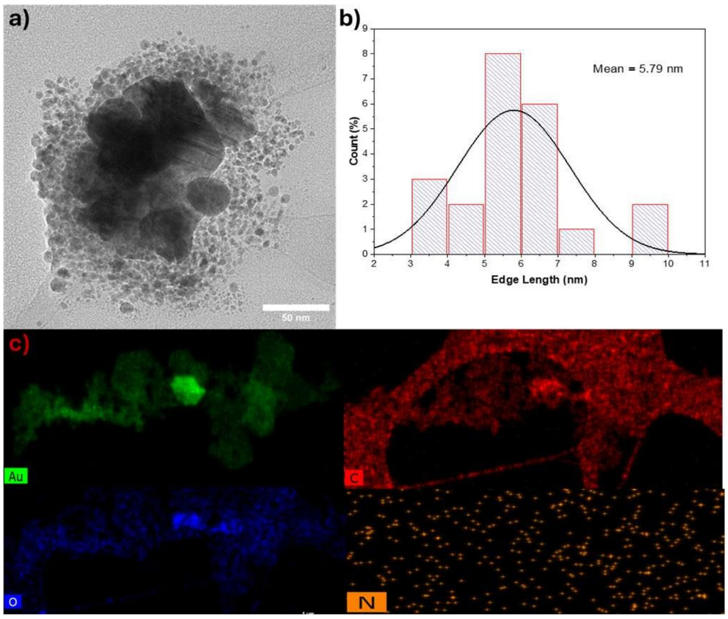

Figure S1. Characterization of AuNCBQD nanoparticles: a) HR-TEM image of AuNCBQD nanoparticles, b) size distribution (histogram) of AuNCBQD nanoparticles, and c) EDS mapping based on Au, C, O, and N within particles.

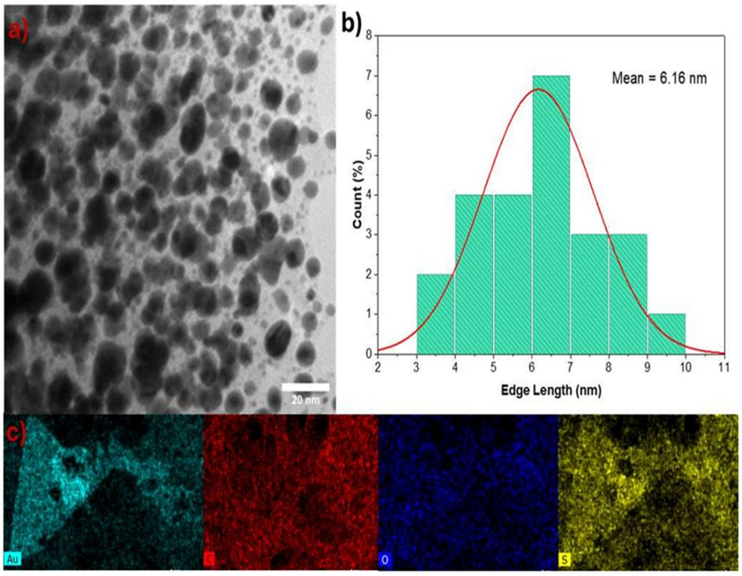

Figure S2. Characterization of AuSCBQD nanoparticles: a) HR-TEM image of AuSCBQD nanoparticles, b) size distribution (histogram) of AuSCBQD nanoparticles, and c) EDS mapping based on Au, C, O, and S within particles.

 https://doi.org/10.3390/pharmaceutics18060647

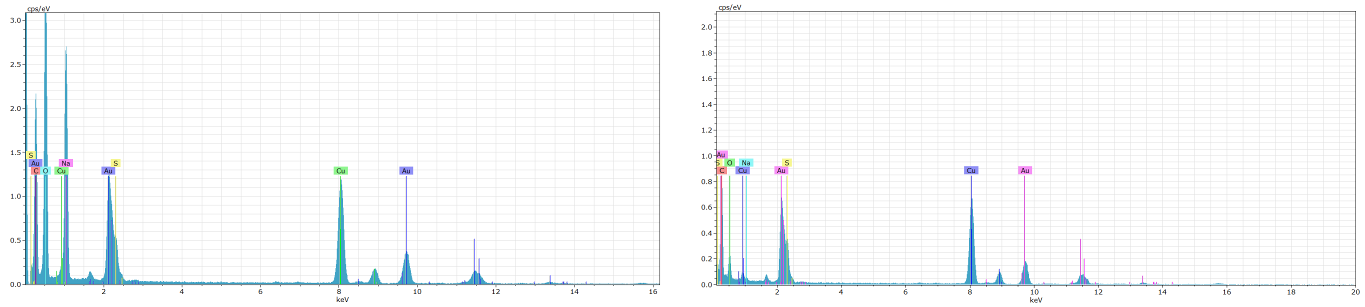

(**a**) (**b**)

Figure S3. EDS mapping of a) AuCBQD particles, and b) AuSCBQD particles.

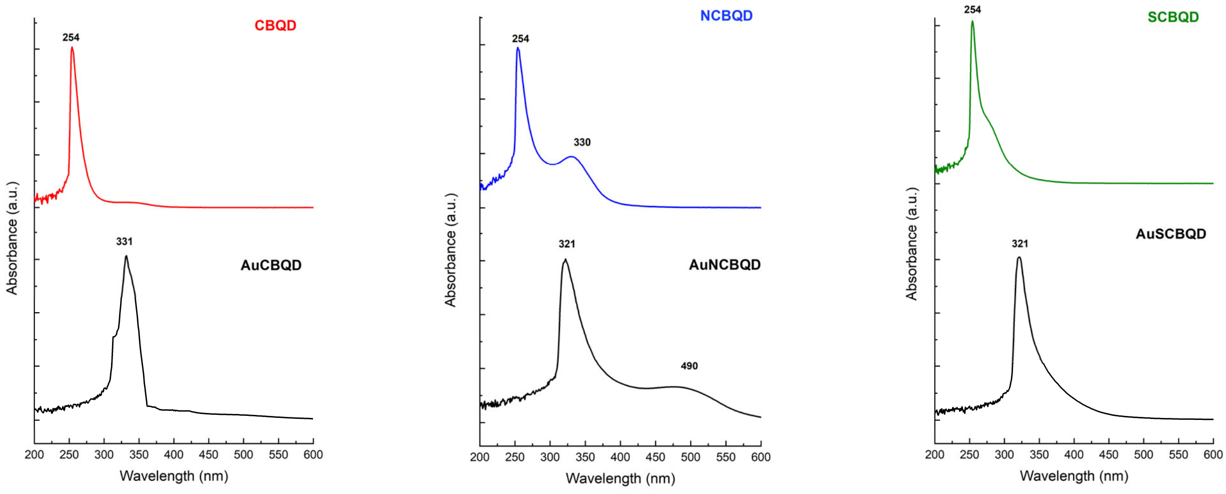

(**a**) (**b**) (**c**)

Figure S4. UV-Vis spectra comparison from bottom to top: a) AuCBQD and CBQD, b) AuNCBQD and N-CQBD, and c) AuSCBQD and S-BQD.

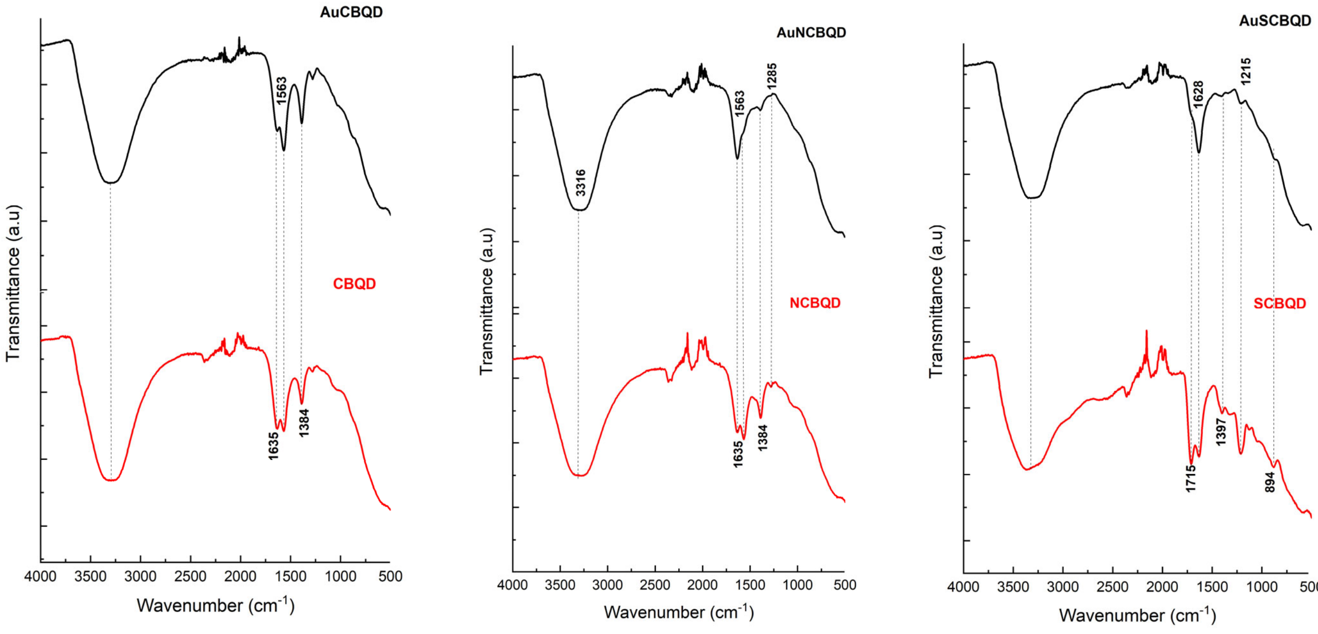

(**a**) (**b**) (**c**)

Figure S5. FT-IR spectra comparison from bottom to top: a) CBQD and AuCBQD, b) N-CQBD and AuNCBQD, and c) S-CBQD and AuSCBQD.

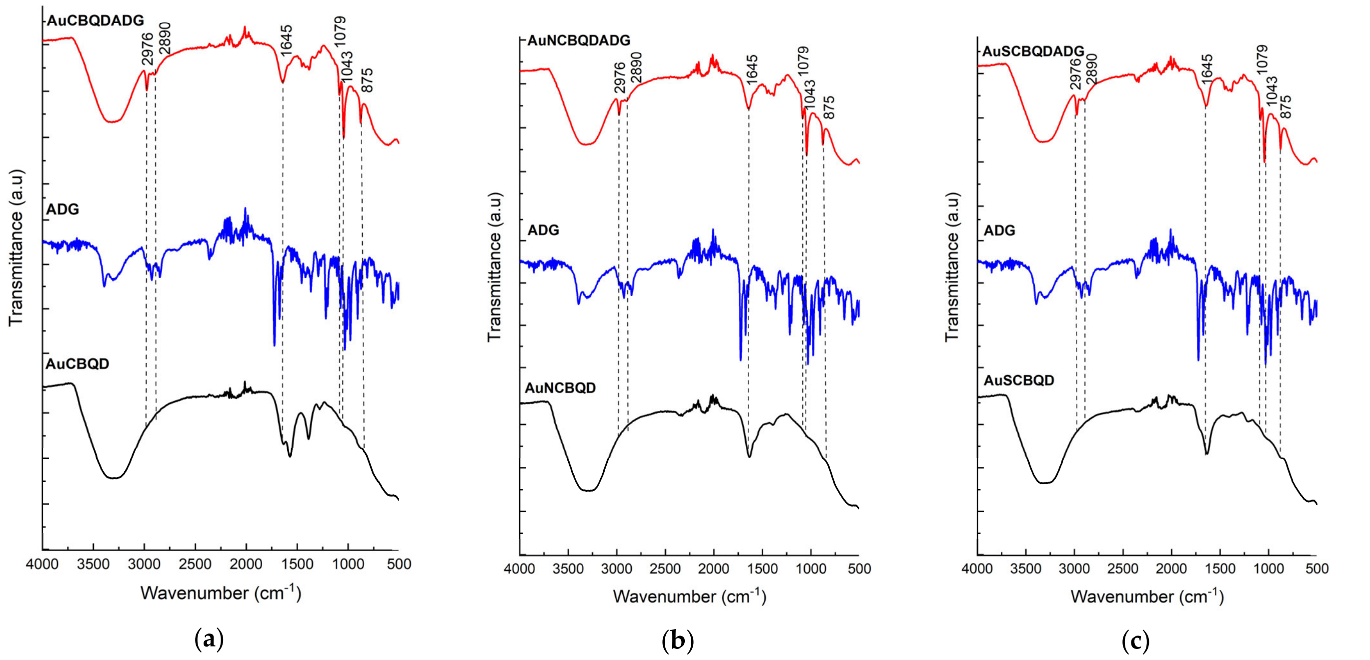

Figure S6. FT-IR spectra comparison from bottom to top: a) AuCBQD, ADG, and AuCBQDADG, b) AuNCBQD, ADG, and AuNCBQDADG, and c) AuSCBQD, ADG, and AuSCBQDADG.

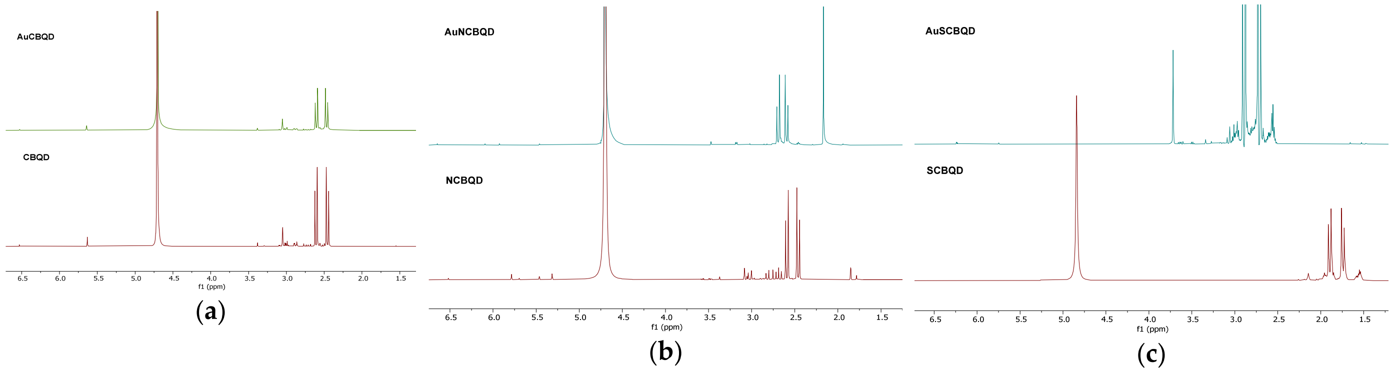

Figure S7. ^{1}H NMR spectra of the different samples from bottom to top: CBQD, D-CBQD, AuCBQD, AuNCBQD, and AuSCBQD.

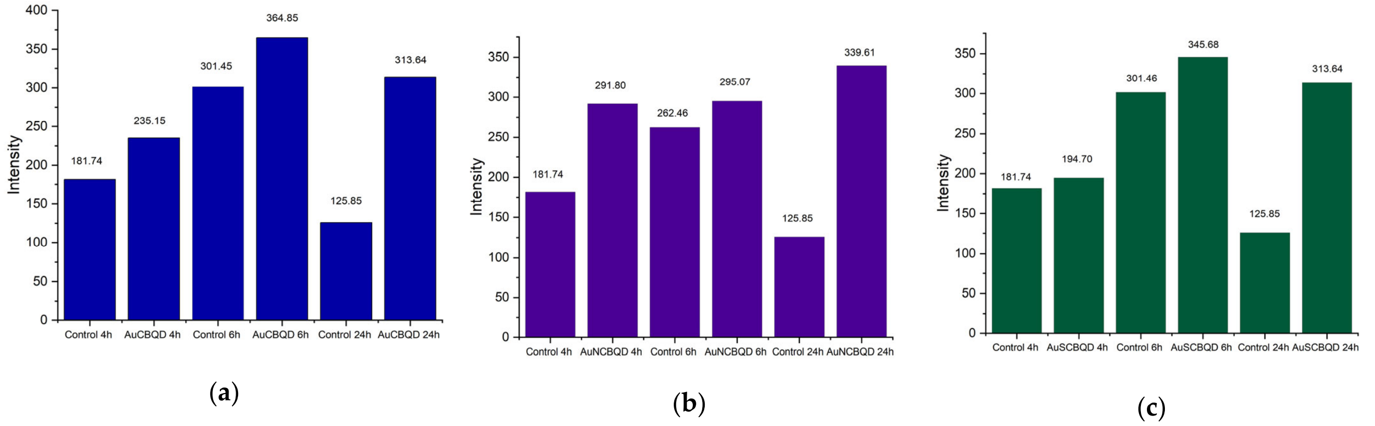

Figure S8. Intensity confocal comparison with the control (PC-3 cells without any material added) at different timeframes (4, 6, and 24 h) in a) AuCBQD, b) AuNCBQD, and c) AuSCBQD.

Table S1. Lack-of-fit test performed for all results in cytotoxicity.

Model	Model Df	RSS	Df	F value	p-value
AuCBQD	278	0.0162	7	2.02	0.0523
AuCBQDADG	278	0.0128	7	1.13	0.3437
AuNCBQD	278	0.0165	7	1.39	0.2079
AuNCBQDADG	278	0.0155	7	1.78	0.0907
AuSCBQD	278	0.0165	7	0.47	0.8505
AuSCBQDADG	278	0.0119	7	1.68	0.1115
ADG	278	0.0501	7	0.51	0.8285

Where: Model Df: Model Degrees of Freedom. RSS: Residual Sum of Squares. Df: Degrees of Freedom difference (compared to the saturated ANOVA model). p-value: Statistical significance; values > 0.05 indicate the model is appropriate.

Table S2. Model performance compared to linear regression using the Akaike Information Criterion (AIC).

Data set	LL.4 Model AIC	Linear AIC
AuCBQD	−1943.86	−80.76
AuCBQDADG	−2008.48	198.98
AuNCBQ	−1938.09	−140.37
AuNCBQDADG	−1956.40	201.73
AuSCBQD	−1938.01	−36.15
AuSCBQDADG	−2029.68	134.64
ADG	−1927.03	208.17

Where LL.4: 4 parameter logistical model. Linear: Linear regression model. Lower AIC values indicate a superior balance between model complexity and statistical fit.

Table S3. Predominant RCBs morphologies after exposure to different concentrations (a = 1.75; b = 8.76; c = 17.9 μg/mL) of gold nanocomposites.

Materials at different concentrations (μg/mL)	Discocytes (%) 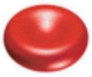	Stomatocytes (%) 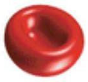	Echinocytes (%) 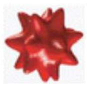	Dumbbells (%) 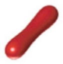	Hemolysis (%)
AuCBQD[a]	8.13	86.33	5.45	0.09	3.02
AuCBQD[b]	7.80	84.73	7.27	0.20	6.73
AuCBQD[c]	7.97	90.06	1.77	0.20	6.09
AuNCBQD[a]	0	98.87	0	1.13	6.09
AuNCBQD[b]	0	99.46	0	0.54	5.83
AuNCBQD[c]	1	98.12	0	0.88	11.69
AuSCBQD[a]	6.02	92.50	1.13	0.17	8.24
AuSCBQD[b]	6.65	92.06	0.54	0.39	3.10
AuSCBQD[c]	6.46	92.61	0.88	0.49	2.26

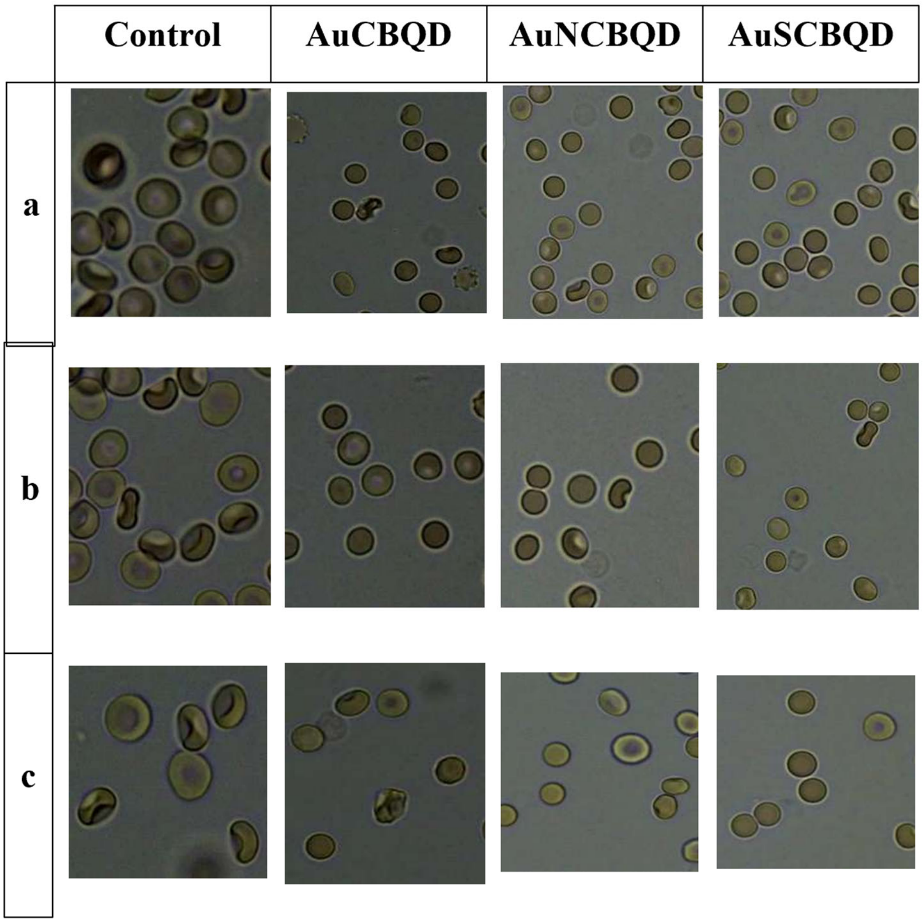

Figure S9. Microscopic images of the RBCs exposed to AuCBQD, AuNCBQD, and AuSCBQD at different concentrations (a=1.75; b= 8.76; c= 17.9 μg/mL) compared to control (RBCs) at the same concentrations.

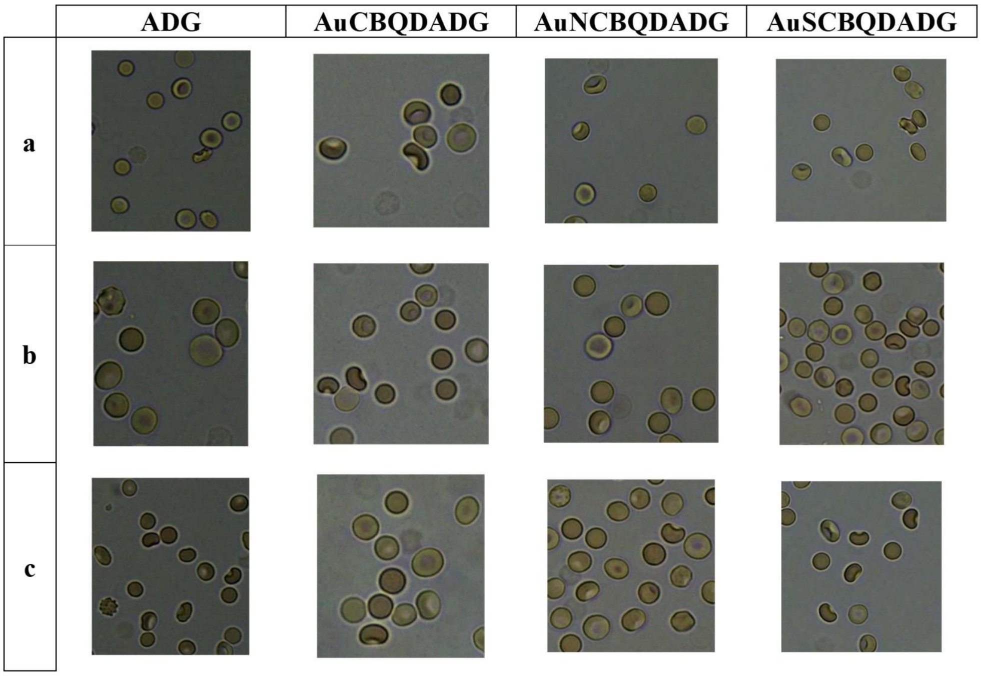

Figure S10. Microscopic images of the RBCs exposed to AuCBQD, AuNCBQD, and AuSCBQD at different concentrations (a = 1.75; b = 8.76; c = 17.9 μg/mL) compared to ADG at the same concentrations.

https://doi.org/10.3390/pharmaceutics18060647
